# Prevention of Transfusion-Transmitted Infections: Dilemmas

**DOI:** 10.3389/fmed.2017.00221

**Published:** 2017-12-12

**Authors:** Hans L. Zaaijer

**Affiliations:** ^1^Sanquin Blood Supply Foundation and Academic Medical Centre, Amsterdam, Netherlands

**Keywords:** blood safety, donor screening, preventive measures, precautionary principle, transfusion-transmitted infection, blood transfusion

## Abstract

To make blood components and blood products safe, many safety measures are applied to avoid transfusion-transmitted infections. Defining a balanced safety policy is not easy, we face several dilemmas: How safe should blood be? Should we opt for maximal or optimal safety? Are perceived threats real and relevant? Should blood be clean while food, air, or mosquitoes are not? Is vCJD still a threat? It seems wise to discuss these issues more in the open.

In developed countries, a variety of safety measures ensures a low risk of transfusion-transmitted infections. These safety measures include donor selection (limiting imported and window infections); skin disinfection and diversion bags (limiting bacterial contamination during blood donation); the screening of donations (enabling timely detection of HBV, HCV, HIV, and *Treponema pallidum*); specific processing (such as leukodepletion, pathogen reduction, and inactivation, which remove or kill certain pathogens); quarantine of plasma (preventing window infections); bacterial culturing (detecting contaminated platelets); and post-donation and post-transfusion notification (respectively, enabling donor- and recipient-triggered look-back procedures, which identify infected recipients). This impressive preventive machinery does not mean that the problem of transfusion-transmitted infections has been solved. New infectious threats arise or are being recognized. Threats may arise locally, such as hepatitis E virus genotype 3 in parts of Europe and Babesia in parts of the USA; or abroad, causing temporary deferral of traveling donors, returning from an ever changing set of affected areas. This dynamic situation necessitates ongoing monitoring, and discussion of safety measures that could be introduced.

Ongoing discussion on another level concerns the notion that existing safety measures may be discriminatory (e.g., asking men-who-have-sex-with-men not to donate) or cost-ineffective (e.g., screening for HTLV after implementation of leukodepletion; or screening for HBsAg after implementation of screening for HBV-DNA and anti-core antibodies). At the same time an entirely new, additional layer of safety is becoming available: pathogen reduction based on photo-chemical treatment of plasma, platelets, or whole blood. Should this technique be implemented on top of existing safety measures, or should pathogen reduction replace (some) existing safety measures? In either scenario: what would be the cost per prevented transmission? Considering the complex mixture of old and new infectious threats, and of old and new safety measures, it is hard to determine at which stage the blood supply is safe enough. Discussing the safety of the blood supply, several dilemmas can be recognized.

## How Safe Should Blood be?

Sometimes clear-cut economic guidelines are applied regarding preventive measures in public health. For example, recently universal vaccination of Dutch children against rotavirus was considered cost-ineffective as long as costs per quality adjusted life year (QALY) gained are more than € 20,000 (1). At the same time, in Dutch blood banking very high costs per QALY occur for HCV and HIV PCR donor screening, which since their start, respectively, in 1999 and 2000, only detected one HCV window infection and no cases of HIV window infection (2). Without thinking, new safety measures may automatically be stacked upon existing safety measures. It must be realized that spending money in blood banking on cost-ineffective preventive measures may be unethical, because the money involved could safe more lives elsewhere in health care. (This line of reasoning must be applied carefully because, for example, any money spent in rich countries would safe more lives when spent in very poor countries.) Advanced pathogen reduction techniques are becoming available. Before these techniques are introduced, it must be analyzed whether and which existing safety procedures can be abolished (3). In addition, the costs of one QALY, gained by pathogen reduction, must be estimated and evaluated using local parameters. Unless these prerequisites are met, it seems wise to withstand the pressure from companies to introduce their pathogen reduction techniques “because they are available.” Complicating an estimation of the cost/benefits ratio is the possibility that pathogen inactivation procedures could harm the functionality of the therapeutic cells or proteins involved.

## Maximum or Optimal Blood Safety?

Among the dilemmas surrounding the prevention of transfusion-transmitted infections, the dilemma whether one can accept a certain residual risk (optimal safety), or whether one should cover each threat (maximum safety), is an easy one. Maximum safety does not exist. Even if all available safety tests and procedures for all agents would have been implemented, one could perform each screening test not once, but twice on each donation, thus increasing the sensitivity of detection. Input volumes for PCR extractions and reactions could be increased, etcetera. Hence, implicitly we always implement a reasonable set of safety measures (optimal safety), not a maximum set of safety measures.

## Is the Threat a Transfusion-Transmitted Threat?

Transfusion-transmitted West Nile virus (WNV) infection can cause serious disease in affected recipients (4). WNV and other arboviruses are on the rise. The Americas were, respectively, invaded by WNV in 1999, by chikungunyavirus in 2013, and by zikavirus in 2015. Worldwide, different serotypes of dengue virus spread into each others areas. Secondary outbreaks of chikungunya and dengue occur in Mediterranean Europe. Recently, in Germany and the Netherlands widespread death occurred in blackbirds and owls, caused by usutuvirus, another arbovirus. Subsequently, usutuvirus viremia has been detected in a German blood donor and in Austrian blood donors. Considering that disease is caused both by mosquito-borne and by transfusion-transmitted WNV, one may assume that all arboviruses cause “their” diseases, whether transmitted by mosquito or *via* transfusion. This assumption may be wrong. The apparent lack of significant disease caused by transfusion-transmitted dengue-, zika-, and chikungunyavirus, even in immunosuppressed patients, suggests that these viruses need transmission *via* a mosquito bite to cause disease. Of course, one must be careful because sound epidemiological data on this topic is scarce. On the other hand, we must realize that tick-bore encephalitis historically seems assumed to be irrelevant for blood safety. Before automatically assuming that zika-, dengue-, chikungunya-, and usutuvirus necessitate blood safety measures like WNV does, we must study the actual threat they pose to blood safety. We must consider to give these viruses the “benefit of the doubt”: as long as significant post-transfusion pathology (in the number of cases and in the nature of pathology) seems absent, blood safety measures may be avoided. In the “post-post-HIV era,” it is too easy to automatically apply the precautionary principle again, which demands that preventive measures should be taken even if cause and effect relationships are not established scientifically (5).

## Should Blood be Clean While Food/Air/Mosquitoes are Not?

In 2009, the third year of the large Q-fever epidemic in the Netherlands, 3 of 1,004 local blood donations were found to be confirmed positive for *Coxiella burnetii* DNA by PCR (6). Should blood donors indeed be screened for *C. burnetti* infection, while nothing was done to decrease the massive exposure of the population (including transfused patients) *via* air? History repeats itself: since July 2017 Dutch blood donations are being screened for the presence of hepatitis E virus, another zoonotic agent. During the first 11 weeks of screening, 48 of 85,023 (1:1,771) donations were found confirmed positive for HEV. We calculated that in Holland only 1 of 700 HEV infections is caused by blood products. In multitransfused at-risk patients, one of 3.5 cases of chronic hepatitis E is caused by blood products (7). Considering the Q-fever and hepatitis E example, one could argue that when “society” accepts large infection pressure *via* common routes such as air and food, it is not necessary to make transfusion safe. On the other hand, it seems that blood transfusion services have the responsibility to provide safe blood to vulnerable patients, even when other significant transmission routes are not eradicated.

## Is vCJD Still a Threat?

Since the outbreak of BSE in Great Britain with subsequent cases of vCJD in humans, many blood banks maintain safety measures to prevent transmission of vCJD *via* blood transfusion and blood products. Examples of safety measures are the exclusion of blood donors who stayed at least 6 months in the UK during 1980–1996, and the exclusion of donors who themselves were transfused. Is it time to lift these restrictions? Until recently, it seemed that the outbreak of vCJD had ended. Unfortunately, in 2016 a new vCJD patient was reported. This patient was found to be heterozygous (methionine/valine) for codon 129 of the human prion gene. This is alarming, because so far all tested vCJD patients were methionine homozygous. Possibly, this first heterozygous patients reflects the start of a second wave of vCJD cases, with longer incubation times than the former homozygous cases. Apparently, people may harbor the infection during many years, and, regarding blood banking, could be seen as asymptomatic, but possibly infectious carriers. On the other hand, no more cases of blood-transmitted vCJD have surfaced. Studies of archived appendices suggest that roughly 1 in 2,500 British appendices tests positive for the vCJD agent. The first and second appendix study involved 12,674, respectively, 32,441 appendices, from persons born between 1961 and 1985, respectively, 1941–1985 (8, 9). These persons experienced dietary exposure to the vCJD agent around 1990; and their appendices were removed in 1995–1999, respectively, 2000–2012; in which 3, respectively, 16 vCJD-prion-positive appendices were found. Is this finding really linked to dietary exposure to the vCJD agent in the 1980s and 1990s, or is there an unrelated, harmless, and natural background presence of the protein in appendices? The Appendix-III study included appendices and persons who did not live in the late 1980s and early 1990s (10). The findings of the Appendix-III study are confusing: possibly the period of dietary exposure is larger than assumed. Alternatively, there may be no connection between appendices testing positive and the BSE/vCJD outbreak. Currently, it seems wise not to abandon vCJD blood safety measures yet.

In summary, several dilemmas exist concerning the desired level of safety of blood transfusion. Perhaps it is good to discuss these issues more in the open, and keep the struggle with difficult and expensive decisions not confined to blood transfusion services and ministries of health.

## Author Contributions

The author confirms being the sole contributor of this work and approved it for publication.

## Conflict of Interest Statement

The author declares that the research was conducted in the absence of any commercial or financial relationships that could be construed as a potential conflict of interest.
